# Hydrolytically Stable Organo-Chemical Surface Functionalization of Bioinert High-Performance Ceramics Enables Osseoconduction and Osseointegration In Vivo

**DOI:** 10.3390/jfb17070348

**Published:** 2026-07-17

**Authors:** Rald V. M. Groven, Changlin Qi, Philipp Schräder, Deniz D. Özman, Lisa Ernst, Zhen Che, Naeem Assasa, Markus Tingart, Frank Hildebrand, Sabine Neuss-Stein, Horst Fischer, Hanno Schenker

**Affiliations:** 1 Department of Orthopedic, Trauma and Reconstructive Surgery, RWTH Aachen University Hospital, Pauwelsstraße 30, 52074 Aachen, Germany; doezman@ukaachen.de (D.D.Ö.); zche@ukaachen.de (Z.C.); tingart@ortho-centrum.de (M.T.); fhildebrand@ukaachen.de (F.H.); hschenker@ukaachen.de (H.S.); 2 Experimental Orthopedics and Trauma Surgery, RWTH Aachen University Hospital, Pauwelsstraße 30, 52074 Aachen, Germany; 3 Department of Dental Materials and Biomaterials Research, RWTH Aachen University Hospital, Pauwelsstraße 30, 52074 Aachen, Germany; pschraeder@ukaachen.de (P.S.); hfischer@ukaachen.de (H.F.); 4 Institute for Laboratory Animal Science, RWTH Aachen University Hospital, Pauwelsstraße 30, 52074 Aachen, Germany; lernst@ukaachen.de; 5 Electron Microscopy Facility, Institute of Pathology, RWTH Aachen University Hospital, Pauwelsstraße 30, 52074 Aachen, Germany; massasa@ukaachen.de; 6 Helmholtz Institute for Biomedical Engineering, Biointerface Group, RWTH Aachen University Hospital, Pauwelsstraße 20, 52074 Aachen, Germany; therapie@neuss-stein.de; 7 Institute of Pathology, RWTH Aachen University Hospital, Pauwelsstraße 30, 52074 Aachen, Germany

**Keywords:** Biological functionalization, implantology, osseointegration, cRGD, mod-HGF

## Abstract

Metal alloys in arthroplasty face limitations including aseptic loosening, material failure, and allergic reactions. High-performance oxide ceramics (HPOCs) represent an alternative but are bioinert, restricting osseointegration and osteoconduction. The aim of this study was to convert bioinert HPOC surfaces into bioactive osteogenic microenvironments through stable peptide-biofunctionalization with cyclic-arginylglycylaspartic acid (cRGD) or modified-hepatocyte growth factor (mod-HGF). In a rabbit femoral defect model, cylindrical ATZ implants were press-fitted into 4.5 × 8 mm defects and examined after 18 weeks. Three groups were compared: uncoated ATZ, cRGD-coated, and mod-HGF-coated implants (*n* = 8 each). Histomorphometry (Movat Pentachrome, H&E, Alcian Blue), scanning electron microscopy, and energy-dispersive X-ray spectroscopy were performed. Both cRGD- and mod-HGF-coated implants significantly increased bone formation and bone–implant contact relative to controls. Reduced fibrotic encapsulation and enhanced vascularization were observed in both groups, with the strongest effects in the mod-HGF group. Microscopy and spectroscopy confirmed greater mineralization and continuous bone–implant interfaces in coated implants. These results demonstrate that biologically functionalizing ATZ with cRGD or mod-HGF effectively enhances bone formation and implant integration in vivo while reducing fibrotic responses. We proved through the in vivo experiments that bioinert HPOCs obtain osseointegrative behavior due to the hydrolytically stable organo-chemical surface functionalization.

## 1. Introduction

Implants used in orthopedic and maxillofacial surgery are frequently made of metallic alloys [1]. However, several limitations to their clinical application exist, such as corrosion, radiopacity, the release of metal ions into the surrounding tissues, stress shielding, allergic reactions, and aseptic loosening [2]. In the field of implantology, high-performance ceramics have demonstrated significant advantages over their metallic counterparts with regard to the aforementioned limitations, and have consequently been the subject of increasing research and clinical application in recent years [3]. Currently, ceramics are used across several fields of musculoskeletal medicine, ranging from, e.g., articulating components in joint arthroplasty to dental abutments and crowns [4,5,6]. The main factors contributing to this increased scientific attention and expanding clinical application are their biocompatibility, low wear abrasion, corrosion resistance, and excellent biomechanical properties for compressive load-bearing [7]. In particular, high-performance oxide ceramics (HPOCs), such as alumina-toughened zirconia (ATZ), are of considerable interest due to their exceptional biomechanical properties as well as their wear and corrosion resistance [8]. The advantage of ATZ over pure zirconia, pure alumina, and zirconia-toughened alumina lies in its exceptional resistance to subcritical crack growth, which contributes to its long-term mechanical reliability under physiological loading conditions [9]. Despite the clear advantages of high-performance ceramic implants made of materials such as ATZ, their inherently bioinert nature remains a significant obstacle that continues to hinder their wider clinical application [10]. In comparison to other bioactive biomaterials, such as tricalcium phosphate, hydroxyapatite, or bioactive glasses, HPOCs do not actively stimulate osseointegration and osteoconduction, thereby limiting their ability to form a robust and enduring biochemical bond with the surrounding bone tissue [10]. Promoting the osseointegrative and osteoconductive characteristics of ceramic implants is therefore of high clinical relevance, both to reduce the risk of implant loosening and to improve the overall treatment outcome. In this context, the biological surface functionalization of HPOC implants may represent a promising strategy to overcome this limitation [11].

In recent years, increasing research has focused on optimizing host–biomaterial interactions through biological surface functionalization, i.e., the application of bioactive protein or peptide coatings to the implant surface [11]. Among these, two particularly promising candidates are cyclic arginyl-glycyl-aspartic acid (cRGD) peptides and modified hepatocyte growth factor (mod-HGF) [12,13]. The cRGD peptide motif mimics integrin receptor ligands and thereby plays a key role in osteogenic differentiation as well as in the attachment of mesenchymal stem cells (MSCs) and osteoblasts to the extracellular matrix (ECM) [14]. Through these mechanisms, it has been shown to promote bone regeneration in both the in vitro and the in vivo setting [15]. Likewise, the multifunctional mod-HGF is involved in several biomolecular processes that are directly relevant to osseointegration and osteoconduction. More specifically, it recruits MSCs and osteoblasts, promotes their osteogenic differentiation, stimulates neovascularization, and modulates ECM remodeling by reducing fibrosis [16,17]. Our group has previously shown that such bioactive peptides can be coupled to HPOC surfaces in a hydrolytically stable manner via a tailored organo-chemical interlayer system consisting of a hydroxyl component and a silane component [18]. Building on this approach, subsequent in vitro studies have demonstrated that this interlayer system results in the strong hydrolytic binding of bone cells to the functionalized ceramic surfaces, underlining its potential as a stable platform for biological functionalization [19]. However, in vivo research on the interaction between surface-coated HPOC implants and the surrounding bone tissue is still largely lacking, which currently hinders the translation of this technology towards potential clinical applications.

The aim of this study was therefore to investigate the impact of organo-chemically and biologically functionalized surfaces of ATZ implant materials on their osseointegrative and osteoconductive capacities in a rabbit bone defect model, comparing three different implant groups: untreated ATZ (control), ATZ biofunctionalized with cRGD, and ATZ biofunctionalized with mod-HGF.

## 2. Materials and Methods

### 2.1. Animal Care and Experimental Groups

This study was reviewed and approved by the responsible German governmental office of animal care and use (Landesamt für Natur, Umwelt und Verbraucherschutz; Landesamt für Verbraucherschutz und Ernährung) under the permit number 81-02.04.2022.A436, and all procedures were performed in accordance with the relevant national and institutional regulations for the care and use of laboratory animals. All sections of this manuscript were prepared in adherence to the ARRIVE guidelines for reporting on animal research [20], ensuring transparent and complete documentation of the experimental design, procedures, and outcomes. Upon arrival at the hosting facility, all animals were examined by a veterinarian to confirm their general health status and were subsequently housed for a period of two weeks prior to the start of the experiments to allow for acclimatization to the new environment. For the experiments, a total of 24 female New Zealand White rabbits, aged 15 to 17 weeks and with a minimum bodyweight of 3 kg, were used (Janvier Labs, Le Genest-Saint-Isle, France).

### 2.2. Implant Coating Preparation

Implant coating was performed according to an established protocol as described in detail by our group before [19,21,22]. ATZ (Ceramys, Mathys Medical, Bettlach, Switzerland) cylinders (Supplementary Figure S1) with a geometry of ⌀ 4.5 × 8 mm were washed three times in 70% ethanol and three times in deionized water (ddH_2_O) in an ultrasound bath. The first group (control group, *n* = 8) consisting of uncoated ATZ cylinders, were packaged and sterilized using 25 kGy gamma radiation (Mediscan).

Subsequently, the remaining implants for the cRGD and mod-HGF groups were hydroxylated for 5 min at room temperature using fresh piranha solution, a 3:1 mixture of sulfuric acid (Sigma Aldrich, St. Louis, MO, USA) and hydrogen peroxide (Merck, Darmstadt, Germany). Implants were then rinsed three times with ddH_2_O, dried overnight and divided in two groups (cRGD and mod-HGF).

The second group (cRGD, *n* = 8) was silanized using 5% 3-Aminopropyldiisopropylethoxysilane (Gelest, Morrisville, PA, USA) in anhydrous toluene (Sigma Aldrich, St. Louis, MO, USA) under a nitrogen atmosphere for 3 h at 120 °C. Subsequently, implants were rinsed three times in technical grade toluene (Merck, Darmstadt, Germany) and ddH_2_O. After drying, the implants were crosslinked by submerging them in 1 mM bis(sulfosuccinimidyl) suberate (Sigma Aldrich, St. Louis, MO, USA) in borate-buffered saline (BBS) for 1 h at 4 °C and rinsed three times in BBS. Then, the implants’ surface was functionalized with cRGD (AS611835, purity > 95%; Thermo Fisher Scientific, Waltham, MA, USA) by submerging them in 1 mM cRGD in BBS solution for 2 h at 4 °C followed by rinsing three times in BBS. Finally, the dried implants were packaged and sterilized using 25 kGy gamma radiation (Mediscan, Kremsmünster, Austria).

The third group (mod-HGF, *d* = 8) was silanized using 5% 3-Mercaptopropyltrimethoxysilan in anhydrous toluene (both Sigma Aldrich, St. Louis, MO, USA) under the same conditions as described above. Modified HGF, engineered to contain an enzymatic tPA-cleavage site and a cysteine binding site, was recombinantly expressed in Flp-In T-REx HEK 293 cells. The protein was recovered from the culture supernatant and purified by immobilized metal affinity chromatography (IMAC) using a 5 mL HisTrap column (HisTrap HP 5 mL, GE Healthcare, Chicago, IL, USA), followed by a heparin column (HiTrap Heparin 5 mL, GE Healthcare, Chicago, IL, USA), on an FPLC system (ÄKTA Purifier, GE Healthcare, Chicago, IL, USA). Purification efficiency of the modified HGF was confirmed by Western blot, and its bioactivity was verified in transwell migration assays using HuH7 cells and human mesenchymal stem cells (hMSCs). Subsequently, implants were crosslinked by submerging them in 20 mM 1,6-bismaleimidohexane (Thermo Fisher Scientific, Waltham, MA, USA) dissolved in dimethyl sulfoxide (Sigma Aldrich, St. Louis, MO, USA) and diluted in PBS for one hour at 4 °C. After rinsing three times with PBS, implant surfaces were functionalized with mod-HGF in PBS for 2 h at 4 °C and rinsed again three times with PBS. Finally, the dried implants were disinfected and packaged in a sterile manner.

### 2.3. Surgical Procedure

**Figure 1:**
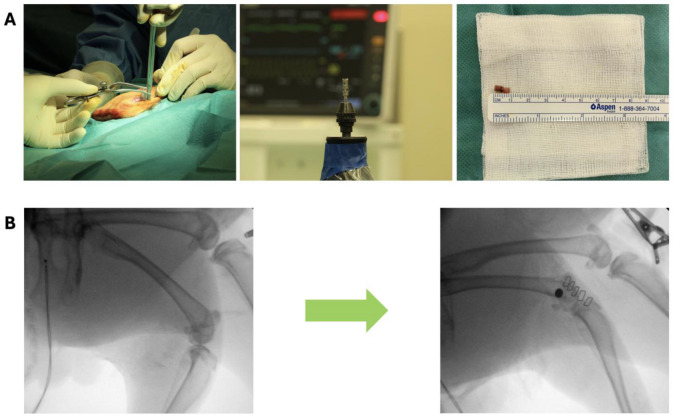
(**A**) Surgical approach and preparation followed by drilling of the bony defect of 4.5 × 8 mm; (**B**) pre- and post-operative X-ray.

Animals were randomly allocated to one of the three groups. Anesthesia was induced via subcutaneous injection of medetomidine (0.1 mg/kgBW) and ketamine (0.2 mL 10%/kgBW). Intravenous access was established via canulation of the marginal ear vein after which animals were orotracheally intubated. General anesthesia was maintained using isoflurane (1.5–3 volume %) and fentanyl (perfusor; 0.01–0.02 mg/kgBW/h). Mechanical, rabbit-adapted volume-controlled ventilation was applied (fraction of inspired oxygen 0.5, tidal volume 10 mL/kg BW, respiratory rate 30–40/min). Prior to surgery, as well as for the first three post-operative days, animals received a single-shot dose of carprofen (4–5 mg/kgBW) and antibiotic prophylaxis (enrofloxacin; 10 mg/kgBW) via subcutaneous injection. The right femur was shaved, disinfected and surgically draped in a sterile fashion. A 4 cm long skin incision was made over the right lateral femoral condyle. Subsequently, the femoral condyle and lateral collateral ligament were exposed through careful preparation and dissection of the overlaying fascia and muscle tissue. Under continuous irrigation with pre-chilled saline solution, a unicortical cylindrical defect of 4.5 mm in diameter and 8 mm of depth was drilled in the right lateral femoral condyle using a trepan drill (Hager & Meisinger GmbH, Neuss, Germany) (Figure 1A). The obtained bony cylinder was removed, and one of three types of implant (CG, cRGD, mod-HGF) was inserted in a press fit fashion. Throughout the whole procedure, the joint capsule and the lateral collateral ligament were preserved. After irrigation of the surgical site, the fascia and subcutis were closed in layers using resorbable sutures, the skin was stapled, and the wound sprayed with silver spray. Post-operatively, implant position was checked using X-ray of the lower extremity in two planes (Figure 1B). The animals were then extubated and monitored in a rabbit intensive care unit, after which they were transferred to their regular housing.

### 2.4. Sample Collection and Preparation

After an observation period of 18 weeks, the animals were euthanized using pentobarbital sodium (2 mL/kg body weight; Narcoren, Boehringer Ingelheim, Ingelheim am Rhein, Germany). Following euthanasia, the right femur was carefully harvested, freed from surrounding soft tissue, and subsequently fixated in 4% paraformaldehyde for a period of six days at room temperature, after which the samples were dehydrated in an ascending ethanol series to gradually remove the tissue water content. Next, the dehydrated samples were embedded in Spurr epoxy resin (Science Services, Munich, Germany) and sectioned at a thickness of 10 µm using laser microtomy technology (TissueSurgeon, LLS ROWIAK GmbH, Hannover, Germany) to obtain undecalcified histological sections for further histological and histomorphometrical analysis.

### 2.5. Histology and Histomorphometry

All samples were stained with Alcian Blue, hematoxylin and eosin, and Movat Pentachrome, and were subsequently digitized using a Fritz slide scanner (PreciPoint GmbH, Garching, Germany) to generate high-resolution images for further analysis. The region of interest (ROI) was defined as a 500 µm zone extending around all borders of the implant, within which direct bone contact and new bone formation were quantified for one representative section of each animal using ImageJ (https://imagej.net/ij/, version 1.54, accessed on 9 August 2025, open source, Bethesda, MD, USA). Furthermore, a qualitative histological evaluation of the stained sections was independently performed by two experienced researchers, who assessed the samples separately in order to minimize observer bias and ensure the reliability of the findings.

### 2.6. Scanning Electron Microscopy and Energy-Dispersive X-Ray Spectroscopy

For scanning electron microscopy (SEM) and energy-dispersive X-ray spectroscopy (EDX), the samples were sputter-coated with a 10 nm carbon layer to ensure sufficient surface conductivity and to prevent charging artifacts during imaging. After coating, the samples were imaged by SEM and analyzed by EDX (Quattro S, Ultradry EDX system, Thermo Fisher Scientific, Waltham, MA, USA) in a high-vacuum environment, using an acceleration voltage of 10 kV at a working distance of 10 mm to allow for both high-resolution morphological assessment and elemental analysis of the bone–implant interface.

### 2.7. Statistical Analysis

Data analyses and graphical representation were performed using GraphPad Prism (Version 10.5.0, GraphPad Software, San Diego, CA, USA). The Shapiro–Wilk test was performed to assess the normality of the data distribution, and group comparisons were subsequently performed using Student’s *t*-test. Data are represented as the mean accompanied by the standard error of the mean (SEM). An α of ≤0.05 was considered to indicate statistical significance for all analyses.

## 3. Results

One animal sustained a right-sided femoral shaft fracture throughout the post-operative course unrelated to the implant, this animal was replaced to keep *n* = 8 in all groups. All other rabbits survived the observation period of 18 weeks. At euthanasia and during sample preparation, no signs of wound dehiscence or other clinical adverse effects around the implant were observed.

### 3.1. Histology and Histomorphometry

**Figure 2:**
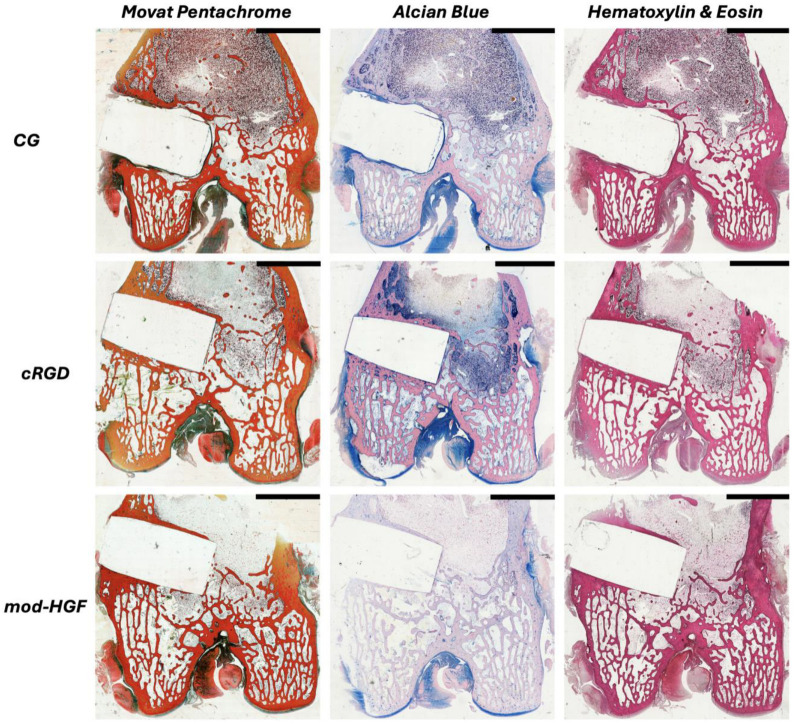
Staining results (Movat Pentachrome, Alcian Blue, hematoxylin and eosin) of the control group (CG) and the two treatment groups; cRGD and mod-HGF. Black scale bars depict 5 mm.

**Figure 3:**
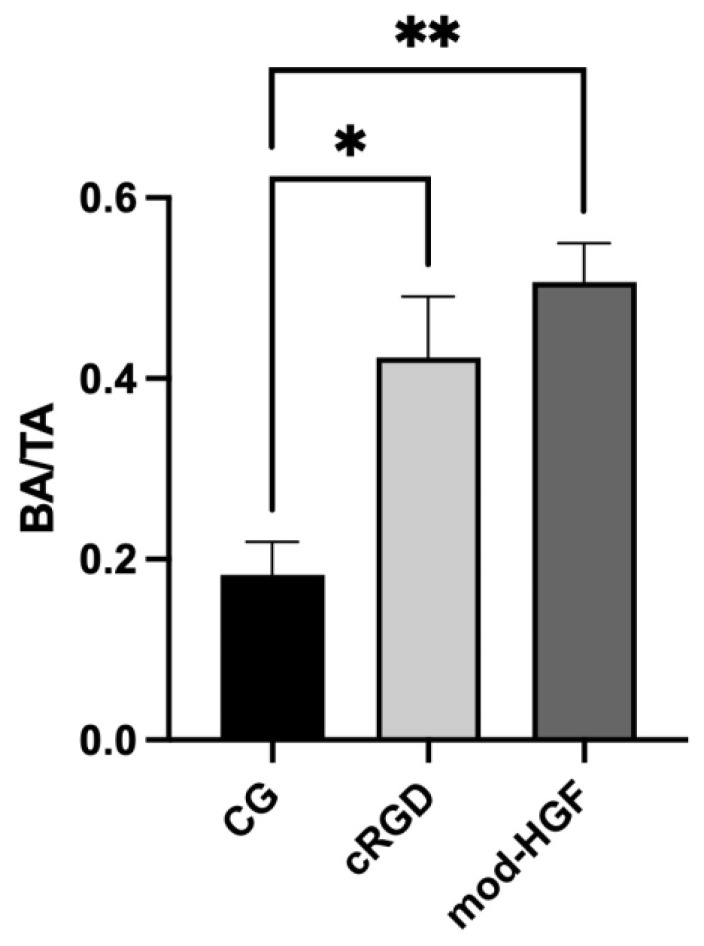
A significant increase in new bone formation (defined as bone area (BA)/total area (TA)) was observed in the region of interest (500 µm around the borders of the implant) in both the cRGD and mod-HGF groups compared to the control group (CG). * *p* < 0.05, ** *p* < 0.01.

Microscopic evaluation of the histological sections revealed no signs of inflammatory cell infiltration or bone necrosis in the tissue surrounding the samples of either the cRGD or the mod-HGF groups, indicating good local tissue tolerance of both functionalized implants. Quantitative histomorphometric analysis demonstrated a significant increase in bone formation, expressed as the ratio of bone area to total area (BA/TA ratio), as well as in bone–implant contact within the region of interest (ROI) of both the cRGD (*p* < 0.05) and mod-HGF (*p* < 0.01) groups compared with the control group (CG) (Figure 2 and Figure 3). Although the difference did not reach statistical significance, a trend towards an increased BA/TA ratio was observed in the mod-HGF group relative to the cRGD group. In the control group, by contrast, a marked increase in fibrotic implant encapsulation was observed, accompanied by significantly reduced bone–implant contact (Figure 2). Furthermore, Alcian Blue staining revealed increased numbers of (hypertrophic) chondrocytes distributed along the borders of the implant in both the cRGD and mod-HGF groups (Figure 2). Lastly, both the Movat Pentachrome and hematoxylin and eosin (H&E) stains showed qualitative increases in neovascularization in the samples from both the cRGD and mod-HGF groups compared with the control (Figure 2).

### 3.2. Scanning Electron Microscopy and EDX Calcium Map

**Figure 4:**
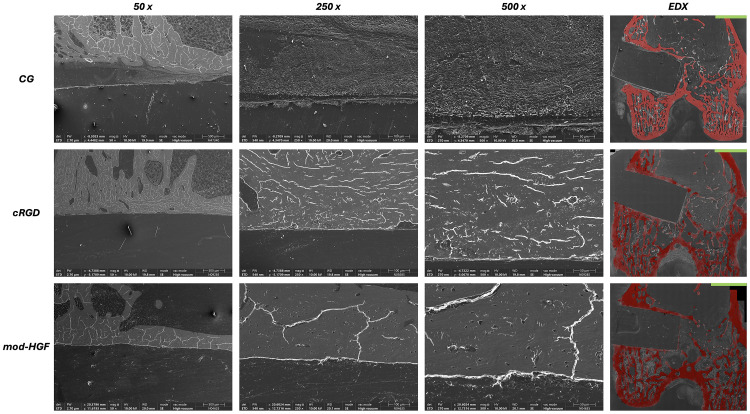
Scanning electron microscopy images of the bone–implant interface at 50, 250, and 500× magnification for the control group (CG) and the two treatment groups; cRGD and mod-HGF. For each group, energy-dispersive X-ray spectroscopy (EDX) was performed; EDX calcium maps are depicted in red, accompanied by green scale bars representing 4 mm.

Scanning electron microscopy (SEM) analysis at 18 weeks revealed distinct differences in the bone–implant interface between groups at the µm-level. In both the cRGD and mod-HGF groups, the interface was characterized by clear bone appositions, with both woven bone and osteoid identified in direct contact with the implant surface (Figure 4). In contrast, the bone–implant interface in the control group was frequently interrupted by gaps or by non-mineralized—predominantly fibrotic—tissue, indicating impaired osseous integration (Figure 4). Complementary elemental analysis using energy-dispersive X-ray spectroscopy (EDX) visualized increased calcium concentrations within the region of interest (ROI) surrounding the cRGD and mod-HGF implants compared with the control group (8.467 × 10^6^ µm^2^, 5.031 × 10^6^ µm^2^, and 2.864 × 10^6^ µm^2^, respectively). The most prominent calcifications were observed in the mod-HGF group, consistent with the SEM findings (Figure 4).

## 4. Discussion

The bioinert nature of ceramics remains an obstacle to their wider clinical application [10]. Alternative bone-repair biomaterials—such as bioactive glasses—achieve osteoconductivity intrinsically through ion release and apatite-layer formation, and continue to be optimized, for example, by rare-earth (Y_2_O_3_) doping to improve mechanical and biological performance [23]. However, such compositionally bioactive materials remain limited by comparatively poor fracture toughness and load-bearing reliability, whereas HPOCs offer excellent biomechanical properties but lack intrinsic bioactivity. Surface biofunctionalization therefore represents a strategy to combine the load-bearing capacity of HPOCs with the osteogenic behavior of bioactive materials. The present study investigated the effect of organo-chemically and biologically functionalized surfaces of HPOC implants on their osseointegrative and osteoconductive capacities in a rabbit bone defect model. The main findings can be summarized as follows:

- Surface coating of HPOC implants with both cRGD and mod-HGF significantly increased bone formation and bone–implant contact compared to the CG;
- Compared to the CG, markedly less fibrotic tissue formation around the implants was observed in both the cRGD and mod-HGF groups;
- At the µm-level, the implants from the cRGD and mod-HGF groups were characterized by predominantly uninterrupted bone–implant bone formation compared to the CG as well as increased calcification around the implant.

Both locally and systemically, no adverse events were observed at any point throughout the entire 18-week follow-up period, indicating good biocompatibility and tolerability of the functionalized implants. Both the cRGD and the mod-HGF surface coatings promoted bone formation and bone–implant contact uniformly around the HPOC implants. Although the difference did not reach statistical significance, the mod-HGF surface coating appeared to be even more effective in promoting bone formation compared with the cRGD coating. This finding offers interesting insights, in particular with regard to mod-HGF, since its role in bone formation and regeneration has been investigated far less extensively than that of cRGD [15,23,24]. In line with our results, cRGD has been shown to promote osteogenic differentiation in both the in vitro and the in vivo setting through enhanced integrin-ligand binding and the upregulation of osteogenic marker genes such as runt-related transcription factor 2, alkaline phosphatase, and osteocalcin [25,26]. In fact, in an in vivo study by Scholz et al., cRGD exhibited effects similar to those of recombinant bone morphogenic protein 2 in an ovine spinal fusion model, with the peptide motif being adsorbed to the cage surface [27]. Although in vitro work has demonstrated that mod-HGF can promote the osteogenic differentiation of MSCs, conclusive in vivo studies are still lacking [28,29]. A murine fracture model by Zhen et al. showed that mod-HGF promoted bone regeneration and neovascularization by enhancing the bone morphogenic protein 2/nuclear factor kappa-light-chain-enhancer of activated B-cells signaling pathway [24]. In contrast, work by Huang et al. demonstrated, in a murine arthritis model, that mod-HGF increased bone resorption by promoting osteoclastogenesis [30]. Taken together, these partly divergent findings highlight that the effects of mod-HGF on bone metabolism may be context-dependent. In the present study, a trend towards increased bone formation was observed in the mod-HGF group compared with the cRGD group.

From an immunological point of view, both compounds are known to elicit anti-inflammatory effects and to promote a tissue-regenerative microenvironment, an observation that is in line with the histological and histomorphometrical findings of the present study [30,31]. Furthermore, qualitative assessment of neovascularization, another key process underlying successful bone formation, revealed that it was enhanced directly around the implant in both the cRGD and mod-HGF groups, albeit to a greater extent in the mod-HGF group. These are interesting findings, particularly against the background that, in both the in vitro and the in vivo setting, cRGD itself does not exhibit direct angiogenic properties and can in fact inhibit endothelial sprouting. This observation suggests that the qualitatively enhanced neovascularization in the cRGD group is most likely a secondary consequence of the osseointegrative effects of cRGD, rather than a direct angiogenic action [32]. In contrast, mod-HGF is a well-established and potent pro-angiogenic mediator, promoting capillary morphogenesis by stimulating endothelial cell proliferation, migration, and survival. This direct angiogenic activity may, at least in part, underlie the increased BA/TA ratio observed in the present study in the mod-HGF group compared with both the cRGD and control groups, as a well-vascularized microenvironment supports the delivery of oxygen, nutrients, and osteoprogenitor cells required for new bone formation [24,33,34].

Our findings should be interpreted in the context of preceding attempts to render bioinert ceramics osseointegrative. Previous strategies have mainly focused on physicochemical or inorganic surface modification. Work by, e.g., Pobloth et al., showed that hydroxyapatite, bioactive glass, and phosphonate coatings improved cancellous osseointegration of zirconia-toughened alumina implants in a sheep model [35], and our own group previously demonstrated that silica-coated HPOCs promote greater ossification than titanium in vivo [7]. Biomolecular functionalization, in contrast, has more often only been evaluated in vitro; RGD-functionalized zirconia, for example, has been shown to enhance osteoblast adhesion but has rarely been assessed in an in vivo setting [36,37]. Where in vivo data for functionalized HPOCs do exist, they have largely relied on the osteoinductive growth factor BMP-2 [38]. The present study extends these previous investigations by directly comparing two mechanistically distinct, covalently and hydrolytically stably immobilized biomolecules—namely, the adhesion peptide cRGD and the pluripotent growth factor mod-HGF—on a single ceramic platform in vivo, and by providing, to the best of our knowledge, the first in vivo evidence of mod-HGF-driven osseointegration on a high-performance ceramic. These findings thereby address an important gap in the current literature and may open new avenues for the biological functionalization of otherwise bioinert ceramic implants.

The present study investigated the effect of biological functionalization of implant surfaces with cRGD and mod-HGF on the bone–implant interaction of HPOC implants. A limitation is the lack of a biomechanical evaluation, which would provide further valuable data in relation to, e.g., implant loosening and load bearing characteristics. Furthermore, this study exclusively used female rabbits, meaning that potential gender-specific differences were not analyzed. However, since rabbits are induced ovulators, hormonal fluctuations are not expected to cause significant bias [39].

## 5. Conclusions

This study demonstrated that the described tailored organo-chemical and biological surface functionalization of HPOC implants with cRGD and mod-HGF is an effective and feasible tool for enhancing osseointegration and consistent with an osteoinductive response—as reflected by de novo bone and osteoid deposition in the functionalized groups—while reducing the local fibrotic tissue response. Both coated active motifs promoted bone formation without adverse effects, with mod-HGF showing a trend toward superior bone formation and regeneration according to the extent of bone formation around the implant. Building on the findings from the present study, several avenues warrant further investigation prior to clinical translation. First, biomechanical testing, such as push-out or pull-out testing, is needed to link the observed histological integration to functional load-bearing stability and resistance to loosening. Second, studies incorporating multiple observation timepoints would clarify the temporal dynamics of osseointegration and osteoconduction, distinguishing early recruitment and differentiation from late-stage remodeling. The present positive findings also warrant for animal models in which load-bearing implant geometries could be applied. From a biomolecular point of view, combinatorial functionalization, pairing the integrin-mediated adhesion of cRGD with the pro-angiogenic and anti-fibrotic actions of mod-HGF, represents a particularly promising strategy to engineer synergistic osteogenic microenvironments. Collectively, these steps could advance biofunctionalized high-performance ceramics toward clinically viable, durably osseointegrating implants.

## Data Availability

The original contributions presented in the study are included in the article, further inquiries can be directed to the corresponding authors.

## Supplementary Materials

The following supporting information can be downloaded at: https://www.mdpi.com/article/10.3390/jfb17070348/s1, Supplementary Figure S1: ATZ (Ceramys, Mathys Medical, Bettlach, Switzerland) cylinders with a geometry of ⌀ 4.5 × 8 mm.

## Abbreviations

The following abbreviations are used in this manuscript:

ATZ	Alumina-Toughened Zirconia
BA	Bone Area
BA/TA	Bone-Area-to-Total-Area Ratio
BBS	Borate-Buffered Saline
BMP-2	Bone Morphogenetic Protein-2
cRGD	Cyclic Arginyl-Glycyl-Aspartic Acid
CG	Control Group
ddH_2_O	Deionized Water
ECM	Extracellular Matrix
EDX	Energy-Dispersive X-ray Spectroscopy
H&E	Hematoxylin and Eosin
HGF	Hepatocyte Growth Factor
HPOC	High-Performance Oxide Ceramic
mod-HGF	Modified Hepatocyte Growth Factor
MSC	Mesenchymal Stem Cell
PBS	Phosphate-Buffered Saline
ROI	Region of Interest
SEM	Scanning Electron Microscopy
TA	Total Area
