# Supplementary material for: Hydrolytically Stable Organo-Chemical Surface Functionalization of Bioinert High-Performance Ceramics Enables Osseoconduction and Osseointegration In Vivo
Source: J Funct Biomater. 2026 Jul 17;17(7):348. doi: 10.3390/jfb17070348 (PMC13413193; doi:10.3390/jfb17070348)
Supplement: Supplementary file 1 [file jfb-17-00348-s001.zip › jfb-4411222-supplementary.pdf]

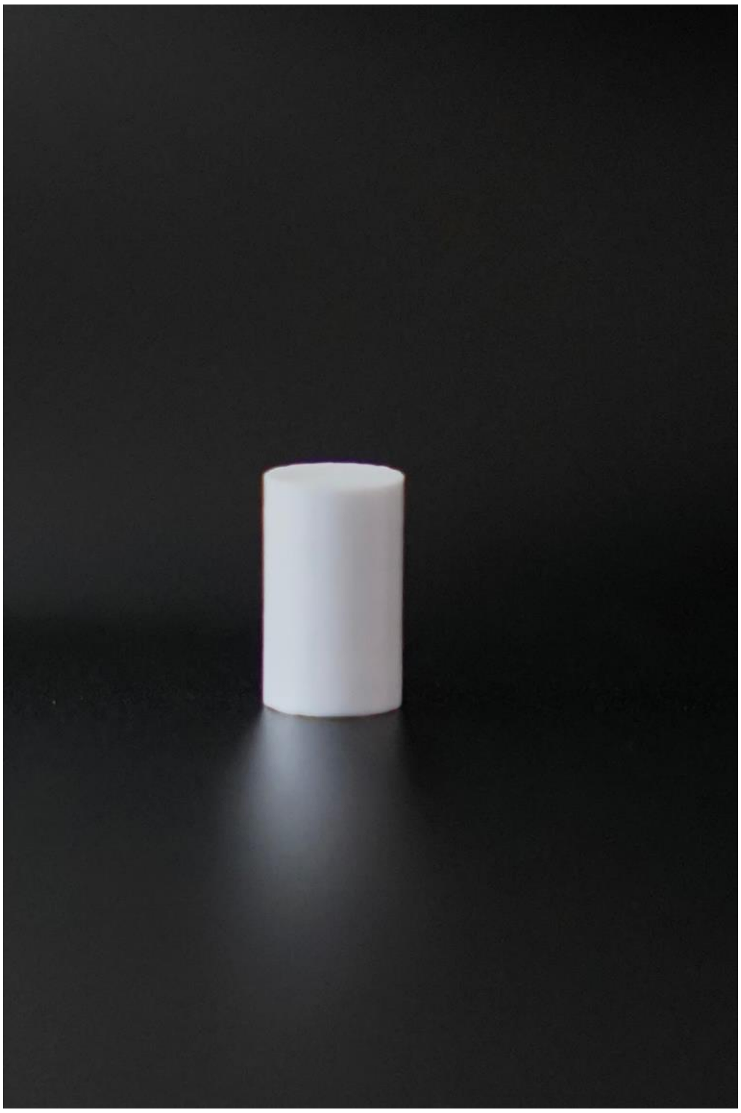

Figure S1. ATZ (Ceramys, Mathys Medical, Bettlach, Switzerland) cylinders with a geometry of 4.5 × 8 mm.
